# P-2093. Persistence of COVID-19 Associated Drug, Alcohol and Firearm Mortality in the United States

**DOI:** 10.1093/ofid/ofaf695.2257

**Published:** 2026-01-11

**Authors:** Roshan Dhand, Kenji Okumura, Kevin Wolfe, Abhay Dhand

**Affiliations:** Emory University, Chappaqua, NY; Westchester Medical Center, Valhalla, NY, Valhalla, New York; Westchester Medical Center, Valhalla, New York; Westchester Medical Center, Valhalla, New York

## Abstract

**Background:**

Population-based deaths in the United States (US) from substance abuse and firearm-related suicides and homicides significantly increased early in the coronavirus disease 2019 (COVID-19) pandemic. Since the mental health impact of various natural disasters have historically outlasted their initial physical impact, we aimed to examine the persistent impact of the COVID-19 pandemic on the change in mortality trends from drug, alcohol, and firearm-related causes. We also aimed to identify the gender, racial, socioeconomic, and regional disparities associated with these mortality trends.

**Figure:**
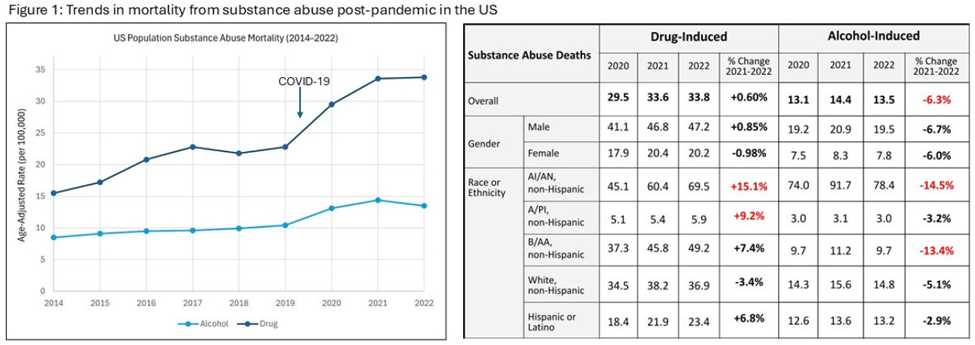

**Figure:**
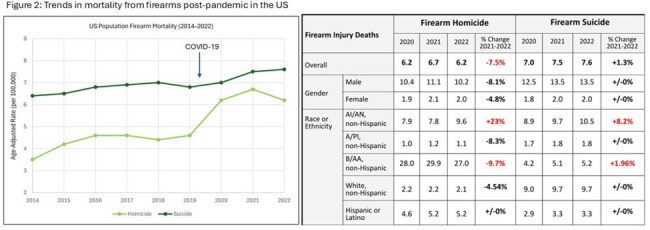

**Methods:**

County-level crude rates of death from 2014–2022 were obtained from the Centers for Disease Control (CDC) WONDER database. Composite indexes of vulnerability like the Community Resilience Estimate (CRE showing the percent population with >3 risk factors) and Social Vulnerability Index (SVI) and other determinants of health were also obtained from various national databases including the US Census. Crude and age-adjusted death rates were calculated per 100,000 population. Pearson correlation was calculated between the county-level data from each index or social determinant and county-level rates of death.

**Figure:**
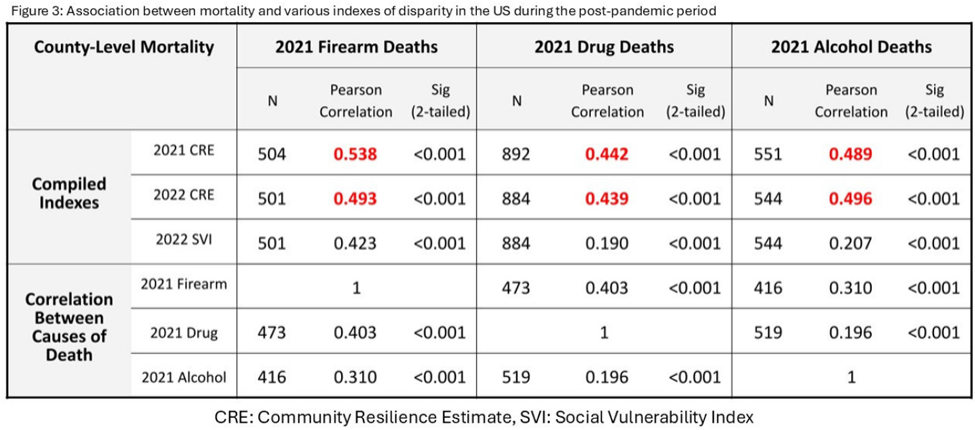

**Figure:**
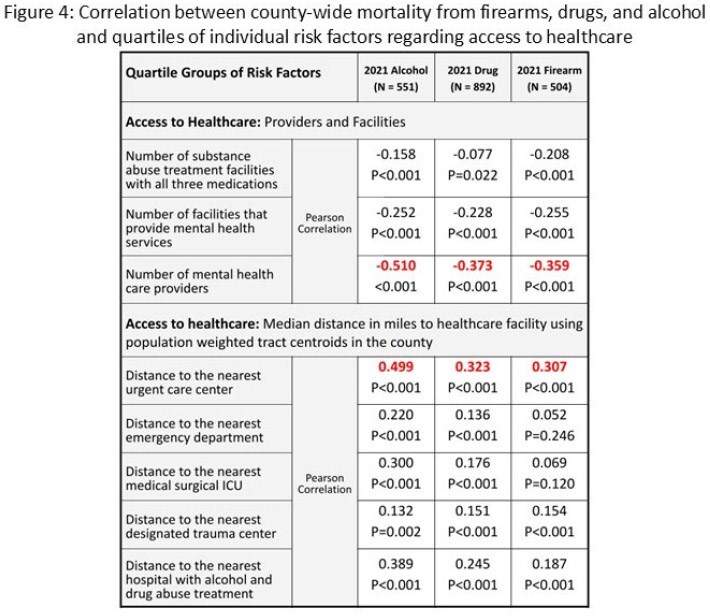

**Results:**

Deaths from alcohol, drugs, and firearms increased from 2014–2021, followed by lower or relatively stable rates from 2021–2022. Alcohol-related deaths decreased across all demographic groups, while drug-related deaths notably increased among American-Indians or Alaska Natives (15.1%). Firearm suicide deaths remained high and stable while firearm homicides decreased across all groups, except for an increase in American-Indians or Alaska Natives (23%). (Figure 1, 2).

The CRE (2021), SVI (2022), and determinants of access to mental health treatment, substance/alcohol rehabilitation, and distance to health care facilities were significantly correlated with local firearm, drug, and alcohol death rates (P< 0.001). (Figure 3,4)

**Conclusion:**

The long-term impact of COVID-19 on population-based deaths can be predicted at local level using various composite indexes and other determinants of health. These tools can then be used to guide future policies and adequate resource allocation in order to mitigate excessive deaths in the post COVID-19 pandemic period.

**Disclosures:**

Abhay Dhand, MD, Eurofins Viracor: Advisor/Consultant|Eurofins Viracor: Honoraria|Merck: Advisor/Consultant|Merck: Honoraria

